# Pelvic physical therapy for male sexual disorders: a narrative review

**DOI:** 10.1038/s41443-025-01034-5

**Published:** 2025-02-27

**Authors:** Emel Sahin, Alma Brand, Elif Nazli Cetindag, Bert Messelink, Hayri Baran Yosmaoglu

**Affiliations:** 1 Physiotherapy and Rehabilitation Department, Faculty of Health Science, Cyprus Aydın University, Girne, Cyprus; 2 Department of Clinical Psychology, Open University, Heerlen, The Netherlands; 3 Urogynecology Doctorate Programme, Graduate School of Health Science, Ankara University, Ankara, Turkey; 4 Department of Urology and Sexology, Pelvic Care Center, Medisch Centrum Leeuwarden, Leeuwarden, The Netherlands; 5 Physiotherapy and Rehabilitation Department, Faculty of Health Science, Baskent University, Ankara, Turkey

**Keywords:** Therapeutics, Quality of life

## Abstract

Pelvic physical therapy is an evidence-based, first-line treatment for many pelvic floor disorders and sexual dysfunction. Studies have shown that pelvic physical therapy programs can both improve pelvic floor dysfunctions and sexual function. This article aims to provide an overview of the current state of the art regarding pelvic physical therapy for male sexual dysfunction to inform healthcare providers who treat men with sexual dysfunction better. A literature review was performed in Google Scholar, PubMed, and Science Direct to find review articles, research articles, and case studies about the effect of pelvic physical therapy treatments for male sexual dysfunction. Twenty-six articles were found about various pelvic physical therapy interventions. Besides this overview of the literature, an overview of interventions used in clinical practice is also provided. This narrative review supports the potential efficacy of pelvic physical therapy in addressing male sexual dysfunction. Pelvic physical therapy approaches that comprise exercise modalities, electrotherapy approaches, manipulative techniques, lifestyle changes, behavioral suggestions, and pain management strategies, should be suggested for potential benefits in improving erectile function, premature ejaculation, and sexual dysfunction-associated chronic pelvic pain. More research is needed to examine the effect of pelvic physical therapy on hypoactive sexual desire and delayed ejaculation.

## Introduction

Sexual dysfunction can be treated by various disciplines [1, 2] including pelvic physical therapists. In pelvic physical therapy (PPT) practice, pelvic floor muscle (PFM) function is a central focus point. Besides in-depth knowledge and skills to rehabilitate PFM function and reduce pain, pelvic physical therapists have profound knowledge of the biomechanics of the lower back, hip, pelvis, and pelvic organs. This knowledge is complemented by skills to treat affected joints, muscles, nerves, and connective tissues in this body region. Notwithstanding this specialized knowledge about the pelvic region, a biopsychosocial therapeutic approach is always required to fully understand the impact of presented symptoms of male sexual dysfunction [3].

Research supporting the effectiveness of PPT in men with sexual dysfunction shows improvements in erectile function, ejaculatory control, and overall sexual satisfaction following a course of pelvic floor rehabilitation [3]. This approach offers a promising alternative or complementary treatment to traditional medical interventions. Because a comprehensive overview of current PPT treatment options for male sexual dysfunction is lacking, this article aims to provide a narrative review of the current PPT approach for male sexual function problems.

## Methods

A literature search for reviews addressing PPT for male sexual dysfunction was performed between January 1993 and December 2023 in Google Scholar, PubMed, and Science Direct using the search terms: male sexual disorders, erectile dysfunction (ED), male delayed orgasm, premature ejaculation (PE), delayed ejaculation (DE), anorgasmia, male orgasm, ejaculation, sexual dysfunction associated chronic pelvic pain syndrome (CPPS), physiotherapy, pelvic floor physiotherapy, pelvic exercise, pelvic physiotherapy, pelvic physical therapy, and exercise. This systematic search focused on sample size, intervention, outcome measures, and results concerning male sexual functioning. In addition, an overview is given of treatment methods based on clinical experience, supported by evidence where this was available.

Twenty-six articles were found specifically on PPT interventions for male sexual dysfunction, including 14 randomised clinical trials and 12 other articles (prospective cohorts and case reports). Questionnaire-based surveys and expert opinions types of articles were also included. Only studies published in English were considered.

## Results

An overview of the literature review is listed in Table 1. Results are presented as well as treatment options and indications of how this option could be beneficial for sexual dysfunctions and CPPS. For interested non-pelvic physical therapist readers, a brief explanation of the working mechanisms is added to increase their level of insight and understanding.

**Table 1:** Overview of the literature search.

(Reference number) Author, Year	Focus	Design	Sample Size (N)	Intervention	Duration of the Intervention	Outcome Measure	Result	Source
Claes & Baert, [42]	ED	RCT	N = 78 (IG) N = 72 (CG) (11 dropouts)	IG: PFMT + Home exrerise CG: Surgery	4 months > 5 sessions	Penile rigidity measured with EMG & ICP	47% at the penile base 34% at the penile tip Cured: 46% Improved: 28% Unchanged: 26%	PubMed ID 8435738
Claes et al. [43]	ED	Prospective cohort	N = 122 (14 dropouts)	PFMT + Home exercise BF	4 months > 20 sessions	ICP	Mild ED group: 52% at the penile base 38% at the penile tip Moderate ED group: 46% at the penile base 37% at the penile tip Cured: 36.1% Improved: 34% Unchanged: 30%	Google scholar
Dorey et al. [44]	ED	RCT	IG: N = 28 CG: N = 27 (22 dropouts)	IG: PFMT + home exercise BF CG: Active lifestyle advice	3 months 5 sessions	IIEF, PIIEF, ED-EQoL, anal pressure	Cured: 40% Improved: 35% Unchanged: 25% (*p* < 0.050)	PubMed ID 15527607
Mohammed et al. [45]	ED	Prospective cohort	N = 40 (4 dropouts) (Chronic obstructive pulmonary disease)	PFMT + Home exercise	4 months 16 sessions	Anal pressure, patient interview for ED	Cured: 35% Improved: 40% Unchanged: 25% (*p* < 0.010)	ScienceDirect
Van Kampen et al. [12]	ED	Prospective cohort	N = 51 (9 dropouts)	PFMT + Home exerecise BF NMES	4 months 16 sessions	Patient interview for ED	Cured: 47% Improved: 24% Unchanged: 12% Dropouts: 17%	PubMed ID 12775199
Kurkar et al. [46]	PE	Prospective cohort	N = 74	PFMT + Home exercise NMES	8 weeks 12 sessions	IELT	Mean (SD): 175 s (46) (*p* < 0.050)	Springer open
La Pera, [9]	PE	Prospective cohort	N = 78	PFMT BF NMES	3 months 20 sessions	IELT PEDT	>10 min Cured: 55% Unchanged: 45%	PubMed ID 25017593
La Pera & Nicastro, [47]	PE	Case series? Prospective cohort?	N = 18	PFMT BF NMES	20 sessions	Patient interview for PE	Cured: 61% Improved: 11% Unchanged:28%	PubMed ID 8699493
Pastore et al. [8]	PE	Prospective cohort	N = 40 (2 dropouts)	PFMT via BF NMES	12 weeks 36 sessions	IELT	Mean (SD): 146 s (38) Cured: 83% Improved: 5% Unchanged: 12% (*p* < 0.050)	PubMed ID 24883105
Pastore et al. [48]	PE	RCT	PFMT: N = 19 SSRI: N = 21 (2 dropouts)	PFMT NMES	12 weeks 36 sessions	IELT	Mean (SD): 126 s (37) Cured: 58% Improved: 11% Unchanged: 26% Dropouts: 5% (*p* < 0.050)	PubMed ID 22320846
Altunan et al. [49]	RRMS	Prospective cohort	N = 9	PFMT	12 weeks	SHIM	SHIM score 17.7 → 16.1 (Not significant)	PubMed ID 33620664
Dorey et al. [50]	ED	RCT	IG: N = 28 CG: N = 27	IG: PFMT BF CG: Home exercise	3 months	IIEF Anal pressure	Cured: 40% Improved: 35.5% Unchanged: 24.5%	PubMed ID 16104916
Lavoisier et al. [5]	ED PE	Observational? Prospective cohort?	ED: N = 122 PE: N = 108	Voluntary contraction coupled + NMES	20 sessions 30 min per session	ICP	Max ΔP increased: ED 87%, PE 88% Max baseline increased: ED 99%, PE 72%	PubMed ID 25082919
Anderson et al. [51]	CPPS + SD	Prospective cohort	N = 133	TPR PRT	8 weeks	PPSS NIH-CPSI	PPSS improved 43% (*p* < 0.001) NIH-CPSI decreased 9.2 (35%) and 7 points (26%) (*p* < 0.001)	PubMed ID 16952676
Begot et al. [52]	ED	RCT	IG = 41 CG = 45 (Acute myocardial infarction)	Home-based walking program	1 month 4 days per week	IIEF	71% decrease in the walking group	PubMed ID 25727080
Carboni et al. [53]	ED	RCT	IG = 11 CG = 11	NMES	4 weeks 15 min. 2 times per week	IIEF EHS	Increase in IIEF and EHS scores in IG	PubMed ID 29785045
Geraerts et al. [54]	ED	RCT	IG = 16 CG = 17 (Radical prostatectomy)	NMES + PFMT	6 weeks 1 time per week + 6 weeks alternating weekly	IIEF	6 out of 14 men in the IG became potent	PubMed ID 26538105
Kałka et al. [55]	ED	RCT	IG = 103 CG = 35 (Ischemic heart disease)	Cycle ergometer Resistance training In- and outdoor exercises	6 months 45 min. per session 5 days per week	IIEF	Better exercise quality with higher exercise tolerance	PubMed ID 23797429
Lamina et al. [56]	ED	RCT	IG = 25 CG = 25 (Hypertensive)	AE (Cycle ergometer)	8 weeks 45–60 min. 3 days per week	IELT	11.50 ± 5.30 vs. 15.14 ± 4.92 pre and post in IG	PubMed ID 19302423
Lin et al. [57]	ED	RCT	IG = 41 CG = 31 (Radical prostatectomy)	PFMT + Home exercise	6–12-months	IIEF	Better scores in IG at 6 and 12 months	PubMed ID 21915042
Palm et al. [58]	ED	RCT	IG = 75 CG = 79 (Ischemic heart disease and/or implantable cardioverter defibrillator)	AE PFMT	12 weeks 60 min. 3 sessions per week	IIEF	Better scores in IG	PubMed ID 30381319
Prota et al. [11]	ED	RCT	IG = 26 CG = 26	BF + Home exercise	12 weeks 30 min. 1 session per week	IIEF	47.1% potency rate in IG	PubMed ID 22573231
Rislanu et al. [18]	ED	RCT	IG = 15 CG = 15	IG: NMES CG: AE	6 weeks 30 min. 2 sessions per week	IIEF	FES group better scores than AE group	PubMed ID 33883841
Yüksel et al. [59]	ED	RCT	N = 60 30: AE 30: PDEI	AE	6 weeks 75 min. 3 days per week	IIEF	Better scores in PDEI group	Science direct
Sighinolfi et al. [13]	ED	Case reports	N = 3 Case reports (Radical prostatectomy)	PFME BF Home exercise	4 months	IIEF	Better scores after intervention	PubMed ID 20092471
Dorey et al. [60]	ED	Case reports	N = 3 men Case reports	1. Lifestyle changes 2. PFME, Manometric BF, Home exercise 3. Home exercise	3 months per intervention	Pelvic floor muscle strength, anal pressure	Better scores after intervention	PubMed ID 12529593

*AE* aerobic exercises, *AUA* american urological association, *BF* biofeedback, *CG* control group, *CP* chronic non-bacterial prostatitis, *CPPS* chronic pelvic pain, *NMES* neuromuscular electrical stimulation, *GTM* global therapeutic massage, *HEP* home exercise program, *ICP* intra cavernous pressure, *IELT* intravaginal ejaculatory latency time, *IG* intervention group, *IIEF* international index of erectile function, *MPT* myofascial physical therapy, *NIH-CPSI* chronic prostatitis symptom index, *PDEI* phosphodiesterase inhibitors, *PE* premature ejaculation, *PEDT* premature ejaculation diagnostic tool, *PIIEF* partner’s international index of erectile function, *PFMT* pelvic floor muscle training, *PPSS* pelvic pain symptom survey, *PRT* paradoxical relaxation training, *PVR* postvoiding residue urine volume, *RCT* randomised control trial, *RRMS* relapsing-remitting multiple sclerosis, *SD* standard deviation, *SEMT* sono-electro-magnetic therapy, *SHIM* sexual health inventory for men, *SSRI* selective serotonin reuptake inhibitor, *Surgery* surgical intervention, *TPR* trigger point release, *VAS* visual analogue scale.

### Pelvic floor muscle exercises

The PFMs play a crucial role in sexual function, supporting erectile function and ejaculation control [4]. Dysfunctional PFMs can lead to difficulties in maintaining an erection or result in PE [5]. By engaging in targeted exercises and therapies, men can optimize PFM function, potentially alleviating symptoms of sexual dysfunction [6]. Therefore, in PPT practice, sexual function needs to be discussed addressing erectile function, ejaculation, and orgasm, including pain before, during, or after orgasm, sexual desire, arousal, and satisfaction.

During sexual activity, relaxation of PFMs is required for penile erection [5]. To sustain an erection when sexually aroused, a subtle coordinated interplay of muscle tension at the base of the penis shaft is needed [2]. Functionally, men need to be capable of contracting and relaxing the PFMs in a coordinated way for optimal sexual function. Any increase or decrease in muscle tension will affect coordination and muscle control. Treatment of any sexual dysfunction in PPT practice will, therefore, always include exercises to optimize muscle relaxation, strength, endurance, and coordination, aiming to regain adequate control over the PFMs and improve sexual functioning [7]. Studies demonstrated that PPT significantly improved erectile function in men with mild to moderate ED [5, 7]. The exercises focused on enhancing pelvic blood flow and muscle function, addressing the underlying causes of ED. PFM training including exercises that improve PFM control can increase intravaginal ejaculation latency time in men with PE [8]. Stronger superficial PFM may enhance orgasmic satisfaction during ejaculation [9]. A systematic review by Gbiri et al. highlighted the positive impact of PFM training on sexual function, concluding that PPT can be considered a valuable non-pharmacological intervention [7].

### Biofeedback treatment

Biofeedback can be useful to provide better insight into PFM function when adequate muscle control is difficult. Visual and auditory feedback during the biofeedback treatment can help to gain insight into the ability to contract or relax the PFM. Biofeedback-assisted exercises may increase PFM control, enhance awareness, and generate faster results, increasing confidence to perform and apply the exercises functionally [10, 11]. Biofeedback measures before and after the treatment will provide the patient with more insight into the treatment results, which can help increase their self-confidence and sense of control over their PFMs and be helpful during the performance of home exercises and applied during sexual activities.

Biofeedback can be an effective non-pharmacological intervention for men with sexual dysfunction. It must be noted that individual responses can vary. Factors such as patient compliance, severity of PFM and sexual dysfunction, and underlying health conditions may influence the effectiveness of biofeedback-assisted interventions. A personalized approach, guided by healthcare professionals, is crucial for optimizing outcomes. Biofeedback-guided treatment can result in greater improvement than PFM exercises alone when treating ED [12].

Other studies demonstrated that biofeedback-assisted exercise interventions may contribute to improved penile blood flow, increased PFM function, and enhanced ejaculatory control in men experiencing ED [11–13]. Biofeedback holds promise as a non-pharmacological intervention for men with sexual dysfunction. However, comprehensive and personalized approaches, along with further research, are essential for establishing the role of biofeedback in the broader spectrum of sexual health interventions.

### Electrostimulation

**Fig. 1:**
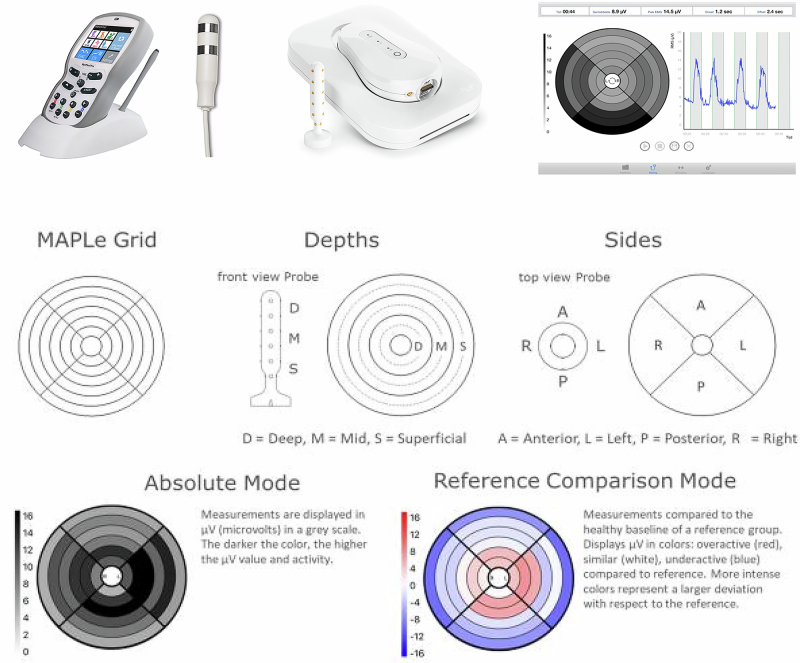
Examples of different biofeedback devices, probes and results of measurements. Top figure (left to right): NeuroTrac Myoplus Pro 4 biofeedback device and intrarectal probe; MAPle biofeedback device and intrarectal probe; An example of EMG measurement results of a patient from MAPle. Middle and bottom figures: Examples of EMG measurement results of a patient from MAPle.

Electrostimulation methods, ranging from transcutaneous electrical nerve stimulation (TENS) to more advanced modalities, have shown promise in addressing underlying factors contributing to sexual dysfunction (see Fig. 1). During electrostimulation, different settings can be applied for different goals, such as improving strength or decreasing muscle tone, and reducing pain [7]. TENS, a non-invasive electrostimulation method, has been investigated for its potential to manage sexual dysfunction [14, 15]. Uribe et al. (2020) show that TENS of posterior tibial nerve when stimulated retrogradely through the stimulation of the posterior tibial nerve, decreases the parasympathetic pathway of the overactive response of the ejaculatory reflex arc thereby contributing to improve lifelong PE [16]. By modulating the ejaculatory reflex, these interventions aim to enhance ejaculatory control. However, research in this area is still in its early stages, necessitating further investigation [15].

The efficacy of electrostimulation methods may vary among individuals, influenced by factors such as the underlying cause of sexual dysfunction, patient compliance, and overall health [17]. A personalized approach, guided by healthcare professionals, is crucial for optimizing outcomes and ensuring patient safety. Nonetheless, electrostimulation presents a promising approach to the management of sexual dysfunction in men [18]. The literature suggests potential benefits in improving erectile function and ejaculatory control [15, 18]. However, further research and clinical trials are needed to establish the efficacy, safety, and optimal use of these methods in diverse patient populations.

### Manipulative therapy techniques

**Fig. 2:**
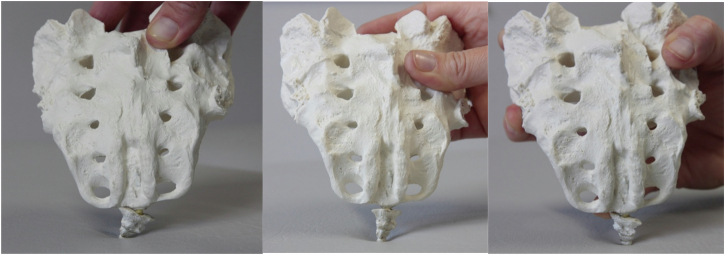
Non-invasive mobilization of the coccyx (NIMOC).

Musculoskeletal complaints can be treated using joint mobilizations and mobilizing exercises to improve the range of motion in the affected joints. Better mechanical movement combined with massaging techniques including fascia and trigger point releases will help to decrease pain and increase the elasticity, contractability, condition, and recovery of affected tissues, improving muscle relaxation, strength, and coordination [19]. Reduced or increased mobility, inflammation, and pain in the lower back, hip, sacroiliac joints, coccyx, symphysis pubis, and surrounding muscles can contribute to pain and discomfort during sexual activity [20]. Sufficient joint mobility also creates more options for different sexual positions [21] (see Fig. 2).

Postural tips and tricks to be applied during sexual activity may help increase sexual pleasure if it leads to a situation in which sexual activities can be performed comfortably and painlessly [2]. When coccyx pain is present, a non-invasive mobilization of the coccyx (NIMOC), can be applied (see Fig. 2). Coccyx pain is often accompanied by increased PFM tone, and decreased coordination. This subtle treatment method has proved very effective in clinical practice [22] and may add to improving sexual dysfunction as a result of pain reduction and increased control over the PFMs.

In men with genital pain or CPPS, increased PFM tone is very common [6, 23]. Tender muscles in the pelvic floor, abdomen, and gluteal region can be detected in men with CPPS [24]. These musculoskeletal complaints are often treated in PPT practice using an integrated multidisciplinary approach including trigger point and myofascial release therapies. Individual responses to manipulative therapy methods can vary, and considerations, such as the underlying cause of sexual dysfunction, patient preferences, and overall health are important. A personalized approach, guided by healthcare professionals, is essential for optimizing outcomes. As research in manipulative therapy for sexual dysfunction evolves, future studies are needed to explore optimal treatment protocols, long-term efficacy, and the integration of manipulative therapies with other interventions to provide a comprehensive approach to sexual health.

### Relaxation and breathing techniques

PFM contraction and relaxation should be coordinated and functional for optimal control over the bladder, the bowel, and sexual function [25]. Therefore, optimizing the PFM function can help to reduce all of these complaints. In the case of increased muscle tone, the first step to focus on is relaxation. PFM relaxation can be pursued by doing local and general relaxation exercises. Adequate local relaxation is easier in combination with deep abdominal breathing techniques, free diaphragmatic movement, and abdominal relaxation [26].

**Fig. 3:**
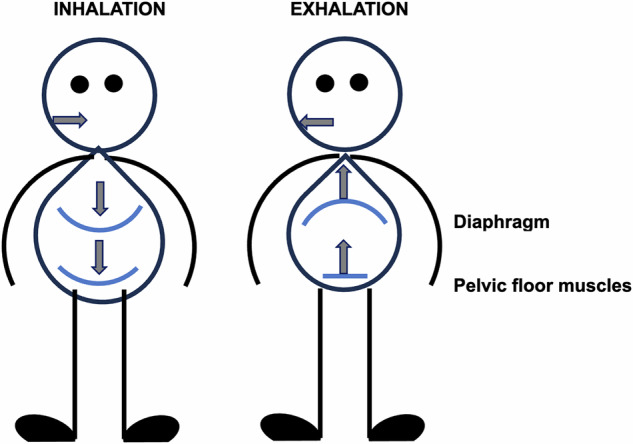
Pelvic floor muscles movement during breathing. During inhalation; diaphragma moves down, this causes your pelvic floor to drop slightly and lengthen. During exhalation, diaphragma rises up, which allows the pelvic floor to move upward with a gentle contraction.

During inhalation, the diaphragm descends pushing the abdominal organs outward into the abdominal wall and down toward the pelvic floor, which ideally follows the downward direction of the moving diaphragm (see Fig. 3). This requires the relaxation of both the abdominal and PFMs. During exhalation, the movement direction is reversed and can be combined with contractions of the pelvic floor and abdominal muscles [26] (see Fig. 3). This procedure can be practiced repeatedly without altering the normal breathing pattern focusing on various levels of contraction and complete relaxation in the process. Better PFM relaxation can create opportunities for a better blood flow toward the penis which may improve erectile function. To allow painless sexual function, the PFMs should relax easily during deep breathing. Very often it is assumed that the PFMs need strengthening. However, it is impossible to adequately contract the PFMs when the muscle tone is increased. Further contraction of the already contracted and tight muscle will be very hard. Therefore, relaxation is very often the first requirement to create movement potential and to feel and coordinate muscle movement because increased PFM tone is common in men with sexual dysfunction, especially in men with CPPS [3, 27].

### Lifestyle changes and physical activity interventions

Healthy living habits positively affect sexual health. Physical therapists encourage physical activity as part of a healthy lifestyle and to prevent illnesses [28, 29]. Physical activity interventions are an essential part of physical therapy to improve overall health and physical capability in ill and healthy people. Regular general exercise reduces fatigue and improves physical fitness, muscle strength, endurance, balance, and flexibility. Therefore, it can be assumed that leisure activities that include physical and systemic exercises and training improve men’s physical condition, which also improves their sexual health [30, 31].

Aerobic exercises, such as walking, swimming, and yoga can also assist PFM coordination as long as the activities are enjoyed and stimulate relaxation [7]. Men who exercise regularly, tend to have higher sexual desire as a result of increased testosterone secretion during the training, which also tends to reduce the occurrence of ED [21].

### Mind-body approaches

Many individuals are unaware of the role their pelvic floor plays in sexual function. By educating men about the importance of these muscles and teaching them how to control and relax them, pelvic physical therapists empower patients to actively participate in their recovery. PPT programs promote awareness of the mind-body connection.

PPT extends beyond physical techniques to include mind-body approaches such as yoga and mindfulness. These practices may positively impact psychological factors contributing to sexual dysfunction, promoting relaxation, and reducing anxiety [32]. Mindfulness approaches, such as mindfulness-based cognitive therapy (MBCT), have shown promise in reducing psychological distress related to sexual dysfunction. Research by Brotto et al. (2008) suggests that mindfulness interventions can enhance sexual well-being by promoting present-moment awareness and reducing performance pressure [33]. Although the participants in this study were females, mindfullness might be considered in men, but further studies are needed.

### Biopsychosocial approaches

Sexual dysfunction in men is a multifaceted issue influenced by biological, psychological, and social factors [34, 35]. Sexual dysfunction, encompassing conditions like ED and PE, demands a holistic understanding that goes beyond purely somatic and physiological aspects. Biopsychosocial approaches recognize the interplay between biological, psychological, and social factors in shaping sexual health [34–36].

Biopsychosocial approaches offer a holistic and patient-centered framework for addressing sexual dysfunction in men. From cognitive-behavioral therapy (CBT) to integrative sex therapy, the literature supports the effectiveness of interventions that consider biological, psychological, and social dimensions [37]. However, ongoing research and clinical exploration are essential to refine and expand these approaches for diverse patient populations.

CBT, a psychological intervention, has demonstrated efficacy in addressing psychological contributors to sexual dysfunction [38]. Studies, such as that by Althof et al. (2006), highlight the role of CBT in reducing performance anxiety and improving overall sexual satisfaction [39]. Integrative sex therapy combines psychological counseling with educational and behavioral interventions [40]. Research by Metz et al. (2017) underscores the effectiveness of this approach in addressing both psychological and relational aspects of sexual dysfunction [38].

Given the diverse nature of sexual dysfunction, a personalized approach that considers individual circumstances, preferences, and the severity of dysfunction is crucial. Physical therapists work closely with individuals to identify specific issues, and contributing factors, and design personalized exercise regimens. PPT should be tailored individually and consist of a biopsychosocial approach to treat effectively sexual dysfunctions that the approaches align with the unique needs of each patient [41].

## Conclusion

This narrative review supports the potential efficacy of PPT in addressing male sexual dysfunction. The effectiveness of PPT for male sexual dysfunction is contingent on a variety of factors, such as the patient’s overall health, adherence to treatment, and the underlying causes of the sexual dysfunction. Therefore, a personalized treatment plan, developed in collaboration between the patient and the healthcare professional, ensures that the specific concerns of each patient are addressed effectively.

Further research and clinical trials can help to establish standardized protocols and to better understand the long-term effectiveness of PPT in this context. More research is needed to examine the effectiveness of PPT in the treatment of hyposexual desire disorder and delayed ejaculation.
